# Synthesis of Brushite Particles in Reverse Microemulsions of the Biosurfactant Surfactin

**DOI:** 10.3390/ijms12063821

**Published:** 2011-06-09

**Authors:** Jyoti Prakash Maity, Tz-Jiun Lin, Henry Pai-Heng Cheng, Chien-Yen Chen, A. Satyanarayana Reddy, Shashi B. Atla, Young-Fo Chang, Hau-Ren Chen, Chien-Cheng Chen

**Affiliations:** 1 Department of Earth and Environmental Sciences National Chung Cheng University, 168 University Road, Min-Hsiung, Chia-Yi 62102, Taiwan; E-Mails: jyoti_maity@yahoo.com (J.P.M.); answer75114@hotmail.com (T.-J.L.); akurireddy@gmail.com (A.S.R.); shashi_org@yahoo.com (S.B.A.); seichyo@eq.ccu.edu.tw (Y.-F.C.); 2 Taipei American School, 800 Chung Shan North Road, Section 6, Taipei 11152, Taiwan; E-Mail: henryc10108230@tas.tw; 3 Department of Life Science, National Chung Cheng University, 168 University Road, Minhsiung, Chia-Yi 62102, Taiwan; 4 Department of Biotechnology, National Kaohsiung Normal University, No.62, Shenjhong Road, Yanchao Township, Kaohsiung County 82444, Taiwan; E-Mail: xavierch2000@yahoo.com

**Keywords:** biosurfactant, reverse microemulsion, brushite, surfactin, nanoparticle

## Abstract

In this study the “green chemistry” use of the biosurfactant surfactin for the synthesis of calcium phosphate using the reverse microemulsion technique was demonstrated. Calcium phosphates are bioactive materials that are a major constituent of human teeth and bone tissue. A reverse microemulsion technique with surfactin was used to produce nanocrystalline brushite particles. Structural diversity (analyzed by SEM and TEM) resulted from different water to surfactin ratios (W/S; 250, 500, 1000 and 40,000). The particle sizes were found to be in the 16–200 nm range. Morphological variety was observed in the as-synthesized microemulsions, which consisted of nanospheres (~16 nm in diameter) and needle-like (8–14 nm in diameter and 80–100 nm in length) noncalcinated particles. However, the calcinated products included nanospheres (50–200 nm in diameter), oval (~300 nm in diameter) and nanorod (200–400 nm in length) particles. FTIR and XRD analysis confirmed the formation of brushite nanoparticles in the as-synthesized products, while calcium pyrophosphate was produced after calcination. These results indicate that the reverse microemulsion technique using surfactin is a green process suitable for the synthesis of nanoparticles.

## 1. Introduction

Nanotechnology has had a great impact on several aspects of the food, drug, and electronic device industries. In recent years, it has been studied extensively in the field of biological mineralization. The majority of bone and teeth tissues naturally consist of calcium phosphate [1,2]. Through many biological and medical studies, it has been found that artificially-made calcium phosphate has characteristics such as biocompatibility and bioactivity, which arise from alteration of its size, morphology, stoichiometry, or the composition of calcium phosphate [3,4]. The general formula Ca_10−_*_x_*(HPO_4_)*_x_*(PO_4_)_6−_*_x_*(OH)_2−_*_x_*, with 0 ≤ *x* < 2, is non-stoichiometric apatite, while calcium phosphates have a Ca/P molar ratio starting from 0.5 [Ca(H_2_PO_4_)_2_] and ending with 2.0 [Ca_4_(PO_4_)_2_O]. Compounds such as brushite and hydroxyapatite have been the focus of many recent studies [5,6]. Calcium phosphate nanoparticles of differing shapes and sizes can be used for different purposes [1,2,5,6].

Sonochemical techniques are used in industrial applications, but these processes are expensive and require complex systems. On the other hand, the microemulsion process is a simple and user-friendly technique that can be used to produce various types of calcium phosphate [7–9]. The main components of the microemulsion process are a mixture of oil, water and surfactant. Additionally, a cosurfactant is used to create a low interfacial tension, which aids in the production of nanosized particles [7]. According to a previous report by Higgins [10], this technique is feasible for industrial applications to produce nanoparticles. As a result, the reverse microemulsion process should be suitable for synthesizing nanoscale brushite calcium phosphates with different morphological structures that contain a water/oil interface in the presence of surfactin.

Surfactin is a type of lipopepetide produced by *Bacillus subtilis* that contains seven amino acids bonded to the carboxyl and hydroxyl groups of a 13–15-carbon acid. It is considered one of the most powerful biosurfactants, as it is able to reduce the surface tension from 72 to 27 mN m^−1^ at a concentration as low as 0.005% [11]. As a biosurfactant, it has the advantages of better biodegradability and environmental-friendly properties.

In this study, brushite nanoparticles of calcium phosphate were prepared through the reverse microemulsion process. Laboratory-made biological surfactin was used at different water/surfactin molar ratios to create different brushite (calcium phosphate) nano-crystal formations. The resulting crystals were then identified and characterized by assessing their structures.

## 2. Results and Discussion

### 2.1. Structural Characterization of Products

**Figure 1:**
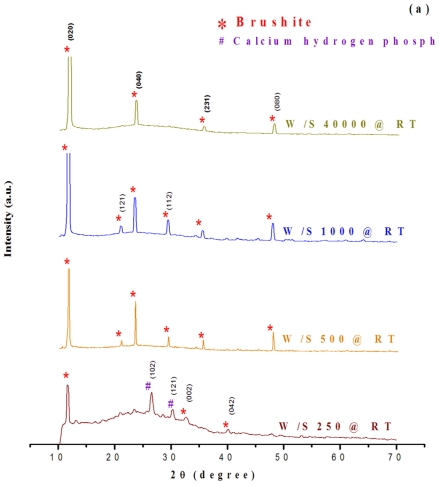

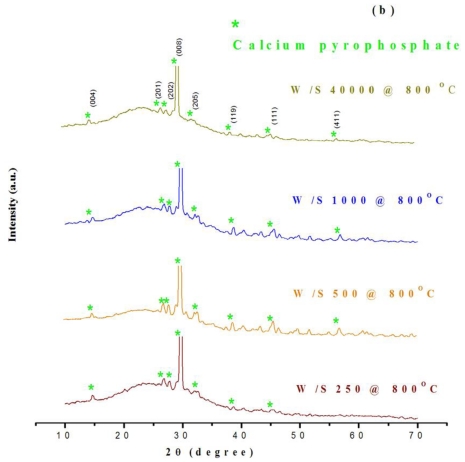
XRD patterns of nanoparticles obtained by the microemulsion process with different water to surfactin ratios (W/S; 250, 500, 1000 and 40,000): (**a**) without calcination, (**b**) with calcination at 800 °C.

The XRD spectra shown in Figure 1 indicate that the maximum peak intensities are at 2*θ* values of around 11.6°, 20.9°, 23.6°, 29.3°, 35.5°, and 48°, attributed to the Miller indices of (020), (121), (040), (112), (231), and (080) of brushite (Figure 1a,b). The spectra confirmed that the products obtained were of unique crystallinity and were mainly composed of the brushite form of calcium phosphate (Figure 1a). The intensity of the diffraction peaks indicated that the particles were fairly well-crystallized. The crystallinity of the particles increased as the W/S ratio of the as-synthesized products increased. In comparison to the spectra of micro-emulsified calcium phosphate, the spectra of the as-synthesized powders with different W/S ratios indicated that brushite was formed in the as-synthesized sets of experiments. However, calcium pyrophosphate (peak at 29.6°) was found in the calcinated products (Figure 1b).

**Figure 2:**
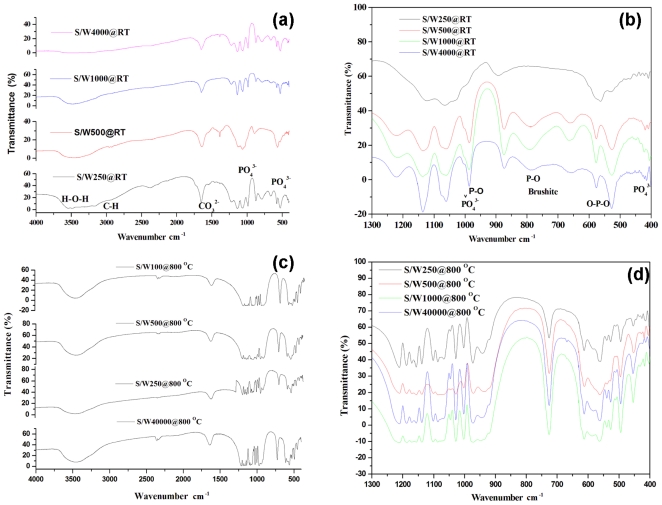
FTIR spectra of nanoparticles obtained by the microemulsion process with different water to surfactin ratios (W/S; 250, 500, 1000 and 40,000): without calcination (**a**) 4000–400 cm^−1^, (**b**) 1300–400 cm^−1^; with calcination at 800 °C (**c**) 4000–400 cm^−1^, (**d**) 1300–400 cm^−1^.

Brushite formation was also confirmed by FTIR spectral analysis (Figure 2). Figure 2a–d show the FTIR spectra of products obtained at different water to surfactin ratios (W/S), in as-synthesized and calcinated forms. The spectra exhibit easily distinguishable bands attributed to PO_4_^3−^ for the non-calcinated form of micro-emulsified calcium phosphate powders (Figure 2a,b). Bands at around 525 cm^−1^ and 575 cm^−1^ were attributed to the *ν*_4_ bending vibrations of the P-O-P mode. Bands at 873 cm^−1^ and 1225 cm^−1^ assigned to the HPO_2_^−4^ group of brushite were observed in all samples. These results were in agreement with the previous reports of Vallet-Regi and Gonzalez-Calbet [12] and Lu *et al*. [13]. A band at 985 cm^−1^ originated from the P-O(H) *ν*_1_ symmetric stretching vibration of PO_3_^4−^. In addition, the peak at around 1060 cm^−1^ was attributed to the *ν*_3_ vibration of the PO_3_^4−^ group. A peak close to 1134 cm^−1^ was assigned to the *ν*′_6_ and *ν*″_6_ of HPO_2_^4−^ ions in brushite. A broad band between 3400–3500 cm^−1^ was observed, due to the stretching (*ν*s) mode of H-bonded OH^−^ or water. A sharp peak in the calcinated product (800 °C) due to P_2_O_7_^4−^ appeared at near 725 cm^−1^ indicating the presence of calcium pyrophosphate (Ca_2_P_2_O_7_) (Figure 2c,d). It was clear that the latter was formed by the loss of one H_2_O molecule from two brushite HPO_2_^4−^ groups under high-temperature conditions. The FTIR spectra thus show that the nano-particles obtained in the as-synthesized samples were brushite, whereas calcium pyrophosphate was obtained after calcination.

### 2.2. Morphological and Particle Size Characterization

**Figure 3:**
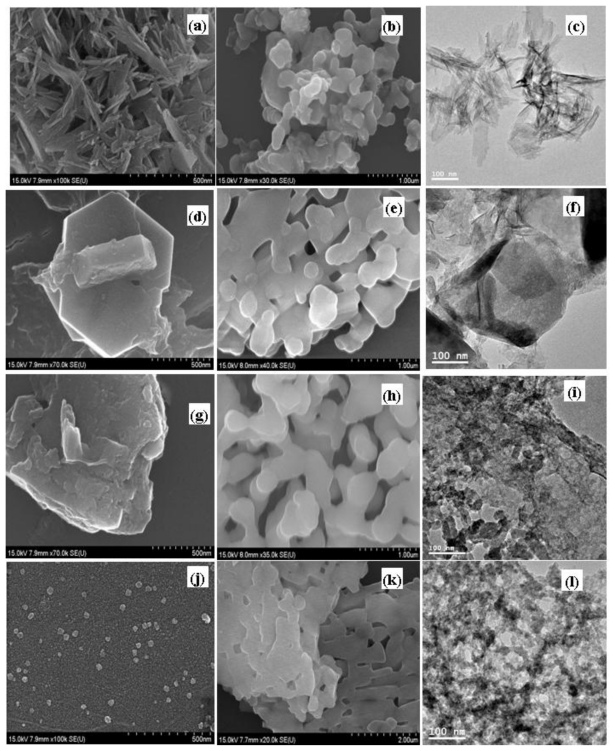
SEM images of nanoparticles obtained by the microemulsion process with different water to surfactin ratios (W/S): (**a**) 250, (**d**) 500, (**g**) 1000 and (**j**) 40,000 without calcination; (**b**) 250, (**e**) 500, (**h**) 1000 and (**k**) 40,000 with calcination at 800 °C. TEM images of nanoparticles obtained by the microemulsion process with different water to surfactin ratios (W/S): (**c**) 250, (**f**) 500, (**i**) 1000 and (**l**) 40,000 without calcination.

Figure 3 shows that the morphology of the particles changed with differing W/S ratios. At a W/S ratio of 250, the particles had an irregular, long needle-like structure (90–200 nm) (Figure 3a–c). A microemulsion at a W/S ratio of 500 produced mixed-type hexagons with a large irregular structure (Figure 3d–f). As the W/S ratio increased further, the inside pool of water was enlarged. The droplet size increased to a much greater dimension, and even deformed the micelle shape. Figure 3j,l shows that an irregular nano-sphere was formed at a W/S ratio of 40,000. However, the calcinated sample induced a recrystallization process to form irregular shapes deviating from spherical shapes and a nanosphere-like structure (Figure 3b) with a diameter of 50–200 nm was observed for the calcinated particles (800 °C) at a W/S ratio of 250. Figure 3b,e,h, and k indicate an increase in particle size after calcination. It was also found that there was an increase in width (irregular plate-like structure) as the W/S ratio increased.

At a W/S ratio of 500, the particle width increased to ~300 nm. The particle shape changed from irregular nano-spheres (200–400 nm) to long aggregated irregular round-edged rods (length 1000–1500 nm) as the W/S ratio varied from 1000 to 40,000. The results suggest that the size of the nanoparticles is mainly controlled by the W/S ratio and reaction temperature. However, the literature reports using P123 and F 127 do not mention any change in the morphology with different surfactant concentration [14]. The results showed that the as-synthesized products were brushite particles and the calcinated (800 °C) products were calcium pyrophosphate particles.

### 2.3. Mechanism of Crystallization in the Reverse Microemulsion Process

Regarding the solubility of calcium phosphate in the crystalline phases of brushite, it was noticed that at a normal pH it precipitates at room temperature. FTIR spectra and XRD analysis indicated that the as-synthesized products obtained at a lower temperature were brushite particles [8]. However, the products obtained after calcination at the higher temperature of 800 °C were calcium pyrophosphate particles [15]. The formation of a solid crystalline phase of calcium phosphate depends on kinetic rather than thermodynamic factors. Recent reports have indicated that the crystallization rate of brushite is much higher than that of HAP at room temperature [8]. The precipitation process of reverse micelles is indicated by the following equation in equilibrium at room temperature:

(1) $${\left({\text{NH}}_{4}\right)}_{2}{\text{HPO}}_{4}↔2{\left({\text{NH}}_{4}\right)}^{+}+{{\text{HPO}}_{4}}^{2-}$$

(2) $${\text{Ca}}^{2+}+{{\text{HPO}}_{4}}^{2-}↔{\text{CaHPO}}_{4}$$

(3) $$\text{Ca}{\left({\text{NO}}_{3}\right)}_{2}\cdot 4{\text{H}}_{2}\text{O}+{\left({\text{NH}}_{4}\right)}_{2}{\text{HPO}}_{4}\to {\text{CaHPO}}_{4}+{\left({\text{NH}}_{4}\right)}_{2}{\text{NO}}_{3}+2{\text{H}}_{2}\text{O}$$

An increase in the crystal growth of brushite is due to the utilization of phosphate ions, which depends on the amount of ammonium ions present in the solution. With an increasing W/S ratio, the number of ammonium ions is increased as a result of (NH_4_)_2_HPO_4_.

The presence of calcium pyrophosphate in a calcinated sample indicates the presence of HPO_4_^2−^. Calcium pyrophosphate is formed by the loss of one H_2_O molecule from two HPO_4_^2−^ groups of brushite under high-temperature conditions. A possible equation of the equilibrium (at 800 °C) according to the results is given below:

(4) $${\text{CaHPO}}_{4}\cdot 2{\text{H}}_{2}\text{O}\to {\text{CaHPO}}_{4}+2{\text{H}}_{2}\text{O}↑$$

The transition of monetite into pyrophosphate occurs at temperatures above 400 °C:

(5) $${\text{2CaHPO}}_{4}\to {\text{Ca}}_{\text{2}}{\text{P}}_{\text{2}}{\text{O}}_{7}+{\text{H}}_{2}\text{O}↑$$

The results of the present study are in agreement with previous reports that indicate that the mass loss at 800 °C produces calcium pyrophosphate through a decomposition reaction [14].

## 3. Experimental Section

### 3.1. Chemicals

The chemical precursors used for the synthesis of nanoparticles were: (i) Ca(NO_3_)_2_·4H_2_O (calcium nitrate tetrahydrate), (ii) (NH_4_)_2_HPO_4_ (biammonium hydrogen phosphate), (iii) surfactin, and (iv) *n*-hexane. All chemicals used were supplied at analytical grade (Sigma-Aldrich, USA), while the surfactin was laboratory-madet.

### 3.2. Calcium Nitrate Tetrahydrate and Ammonium Phosphate Solutions

Two solutions were made for the microemulsion (Table 1). The first solution contained Ca(NO_3_)_2_·4H_2_O (2.36 g) and water (100 mL). The second solution contained (NH_4_)_2_HPO_4_ (1.584 g) and water (100 mL). Both solutions were stirred separately for 5 min.

### 3.3. Surfactin Production

*Bacillus subtilis* (BBK006) was used to produce surfactin. The microorganism was cultured in M9 medium (1 g/L NH_4_Cl, 3 g/L KH_2_PO_4_, 6 g/L Na_2_HPO_4_, 5 g/L NaCl, 1 mmol/L MgSO_4_, and 0.1 mmol/L CaCl_2_) at 37 °C for sufficient growth. Afterwards, the bacteria and supernatant were separated using high-speed centrifugation at 13,000 rpm for 30 min (Himac CR-22G, R20A2Rotor, Hitachi, Japan). The supernatant was acidified to pH <2 using hydrochloric acid. Surfactin was collected using high-speed centrifugation at 13,000 rpm for 30 min. The precipitated surfactin was then dried to surfactin powder using a freeze drier (Biotron 3180C).

### 3.4. Synthesis of Nanoparticles by the Reverse Microemulsion Process

The reverse microemulsion process was used to prepare calcium phosphate nanoparticles. Surfactin was added to produce an emulsion layer that acted as a potential reaction space. The ratio of water to surfactin (W/S) used varied from 250 to 40,000 (250, 500, 1000 and 40,000, Table 1). Calcium nitrate tetrahydrate (2.25 mL, 0.2 M) and ammonium phosphate (0.12 M) were separately added to 10 mL *n*-hexane in two centrifuge tubes “A” and “B”. Different amounts (g dry weight) of surfactin (Table 1) were then added. These two solutions were mixed together by rigorous shaking. After 4 days of reaction, a white product was formed and was collected by centrifugation, washed repeatedly with methanol, and dried overnight at 50 °C. All synthesized powders were calcinated at 800 °C for 3 h. The calcinated powders were labeled W/S250@800 °C, W/S500@800 °C, W/S1000@800 °C and W/S40000@800 °C, and the synthesized powders were labeled W/S250@RT, W/S500@RT, W/S1000@RT and W/S40000@RT.

### 3.5. Characterization of Particles

#### Morphological study by TEM and SEM

The powders were dispersed in ethanol before analysis. The morphology of the synthesized powders was characterized by TEM (JEOL JEM 2010, Japan) using an accelerating voltage of 200 kV. The powders were also analyzed by SEM (Hitachi S4800, Japan) with a coating (Pt) operated at 0.1–30 kV.

#### Assessment of functional groups by FTIR

The compositions of the synthesized powders were identified by FTIR (Jasco FT/IR-460 plus) with the assistance of KBr. The functional groups of the samples were observed in the spectral region of 4000~1300 cm^−1^ and the fingerprint region of 1300~400 cm^−1^.

#### Assessment of phase composition and crystallinity using XRD

The phase composition and crystallinity of both the calcinated and non-calcinated powders were analyzed by X-ray diffraction (LabX XRD6000) with CuKa radiation (λ = 0.15418 nm) at 40 kV and 30 mA. The angle was set to 10–70 degrees, four degrees per minute.

## 4. Conclusions

It has been shown that microemulsions created by surfactin successfully produced calcium phosphate of unique shapes and sizes. The reverse microemulsion process resulted in the formation and growth of brushite nanoparticles under non-calcinated conditions at room temperature. On the other hand, calcium pyrophosphate particles were formed under calcinated (800 °C) conditions. The crystalline structure of the calcium phosphate depended on the ratio of the calcium nitrate tetrahydrate and ammonium phosphate water solution to surfactin. The different structures obtained included hexagonal-shaped, thin-layered, needle-shaped, and small roundish-shaped crystals. All the particles produced were nano-sized (16–200 nm), and the technique is inexpensive and eco-friendly. This novel approach can potentially be used in a wide range of technological applications.

## Figures and Tables

**Table 1:** Recipe for reverse microemulsion with a water/surfactin ratio (W/S) from 250 to 40,000.

Water/Surfactant Ratio (W/S)	A			B		

	Calcium solution (mL)	*n*-hexane (mL)	Surfactin (g)	Phosphate solution (mL)	*n*-hexane (mL)	Surfactin (g)
250	2.25	10	1.0320	2.25	10	1.0320
500	2.25	10	0.5160	2.25	10	0.5160
1000	2.25	10	0.2580	2.25	10	0.2580
40,000	2.25	10	0.0065	2.25	10	0.0065
